# Supplementary material for: Contribution of dorsal horn CGRP-expressing interneurons to mechanical sensitivity
Source: eLife. 2021 Jun 1;10:e59751. doi: 10.7554/eLife.59751 (PMC8245130; doi:10.7554/eLife.59751)
Supplement: Figure 6—source data 1. — Most CGRP-tdTomato neurons showed delayed firing patterns (delayed 19, tonic 1, reluctant 2, single 2, no response 3). Based on electrical stimulation of dorsal roots, we conclude that CGRP interneurons in the lumbar cord predominantly receive monosynaptic input from Aβ primary afferent fibers. [file elife-59751-fig6-data1.docx]

**Figure 6 – data source. Table of intrinsic properties of CGRP-tdTomato neurons**

***mean +/- SD**

| **Membrane properties** | Spinal cord | Caudalis |
| --- | --- | --- |
|  | 22 cells, 8 mice | 5 cells, 2 mice |
| Vm (mV) | -78.9+/- 7.7 | -76 +/- 2.8 |
| Rheobase (pA) | 62.7 +/- 40.8 | 50.52 +/- 26.13 |
| AP thresh. | -41.3 +/- 15.2 | -48.15 +/- 9.44 |
| Cm (pF) | 40.3 +/- 14.7 | 44.55 +/- 12.63 |
| Rm (mOhm) | 603.1 +/- 296.8 | 710 +/- 463 |
|  |  |  |
| **Firing pattern** |  |  |
| Delayed | 17/22 | 2/5 |
| Tonic | 1/22 | 0 |
| Reluctant | 1/22 | 1/5 |
| Single | 0 | 2/5 |
| No response | 3 | 0 |
|  |  |  |
| **Afferent input** | 5 cells, 3 mice | n/a |
| A-mono | 5/5 | n/a |
| C-poly & mono | 2/5 | n/a |
